# Lipoleiomyoma: a potential mimic of an ovarian dermoid cyst on MRI—a case report

**DOI:** 10.1093/jscr/rjae041

**Published:** 2024-02-10

**Authors:** Fatima El Hassouni, Soumaya El Graini, Samia Sassi, Siham El Haddad, Lamiaa Rouas, Mounia El Youssfi

**Affiliations:** Department of Obstetrics and Gynecology, Oncology and High-Risk Pregnancies, Maternity Hospital Souissi, Ibn Sina University Hospital, Rabat 10100, Morocco; Mohamed V University, Rabat 10000, Morocco; Radiology Department, Children’s Hospital, University Mohammed V, Rabat 10100, Morocco; Mohamed V University, Rabat 10000, Morocco; Department of Pathology, Ibn Sina University Hospital, Rabat 10100, Morocco; Mohamed V University, Rabat 10000, Morocco; Radiology Department, Children’s Hospital, University Mohammed V, Rabat 10100, Morocco; Mohamed V University, Rabat 10000, Morocco; Department of Pathology, Ibn Sina University Hospital, Rabat 10100, Morocco; Mohamed V University, Rabat 10000, Morocco; Department of Obstetrics and Gynecology, Oncology and High-Risk Pregnancies, Maternity Hospital Souissi, Ibn Sina University Hospital, Rabat 10100, Morocco; Mohamed V University, Rabat 10000, Morocco

**Keywords:** uterine lipoleiomyoma, cartilaginous metaplasia, adnexal mass, diagnostic imaging

## Abstract

Uterine lipoleiomyomas are rare variants of uterine leiomyomas which is composed of adipocytes and smooth muscle cells. In this report, we describe the case of a 39-year-old patient who presented with persistent, isolated pelvic pain. Ultrasonography showed an oval, well-defined left ovarian mass. Computed tomography (CT) scanning showed a predominantly-fatty mass with tissular components, no calcifications and heterogeneously enhanced after injection, suggesting initially a mature teratoma. Magnetic resonance imaging (MRI) findings revealed a latero-uterine mass, suggesting the presence of a left ovarian dermoid cyst with a potentially-malignant fleshy component. A subsequent pathology report revealed a lipoleiomyoma with cartilaginous metaplasia. Most notably, despite the fatty nature of the tumour and the use of MRI, the pedunculated appearance of the lipoleiomyoma observed intraoperatively mimicked a dermoid tumour even on imaging. Improved understanding of leiomyoma variants and secondary degenerative changes can help prevent misdiagnosis.

## Introduction

Leiomyomas are the most common benign gynaecologic tumours with a prevalence ranging from 25 to 40% [1]. Leiomyomas consist of smooth muscle cells that arise from the myometrium. Though rare, leiomyomas may contain heterologous elements such as fatty tissue, skeletal muscle, chondroid and osseous tissue [2].

A lipoleiomyoma is a rare variant of uterine leiomyomas. It was first described in 1965 by Jacobs and has been characterized as being composed of varying amounts of adipose tissue and smooth muscle cells [3]. The reported incidence rate of uterine lipoleiomyomas ranges from 0.03 to 0.2% of all uterine leiomyomas [4].

Cartilaginous metaplasia of soft-tissue leiomyomas is common, but uterine leiomyomas with cartilaginous metaplasia occur very rarely [2].

## Case report

A 39-year-old woman, gravida 3, parity 2, with no medical or surgical history of note and a normal menstrual cycle was admitted to our hospital’s gynaecology ward with complaints of isolated pelvic pain. Physical examination revealed a uterus of normal size with a left latero-uterine mass, separated from the uterus. The serum level of cancer antigen-125 was normal.

**Figure 1:**
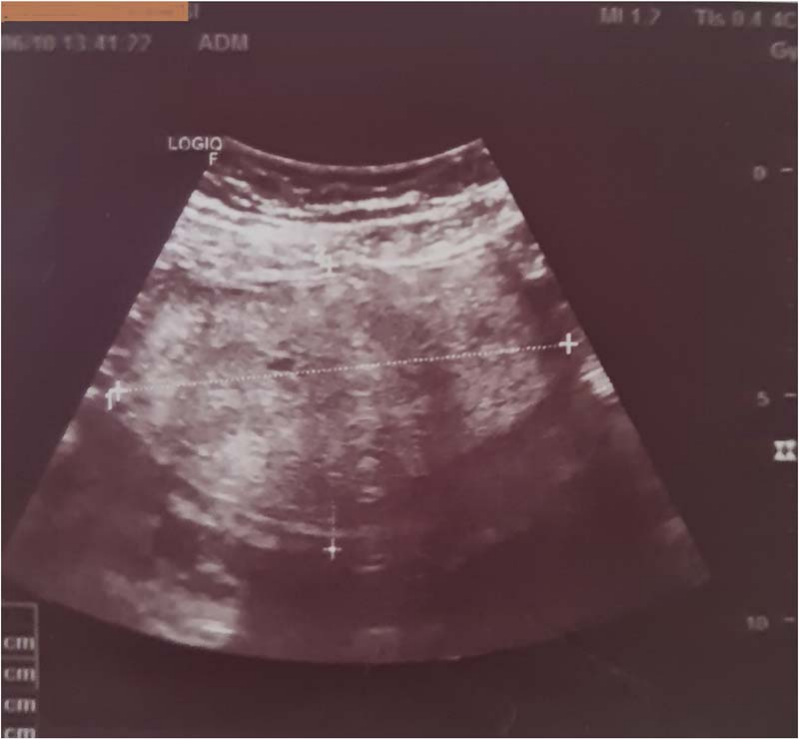
Ultrasound showing an oval, well-limited left ovarian mass, with regular contours, and a heterogeneous, hyperechoic echostructure and cystic areas.

Subsequent pelvic ultrasound showed an oval, well-defined left ovarian mass, with regular contours, and a heterogeneous, hyperechoic echostructure with cystic areas, and did not show any vascularity on colour Doppler imaging (Fig. 1).

CT showed a predominantly-fatty mass with tissular components and no calcifications, and heterogeneously enhanced after injection, suggesting initially a mature teratoma.

**Figure 2:**
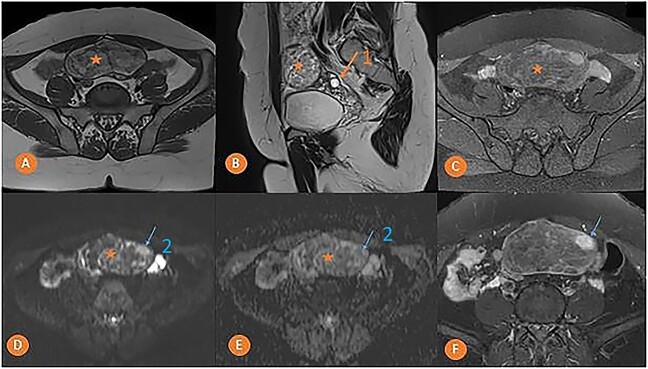
Pelvic MRI in axial T1 (A), sagittal T2 (B), axial T1 FS (C), axial DWI (D) with ADC mapping (E), and axial T1 injected (F) slices showing a median pelvic mass (star) lateralized on the left in contact with the homolateral ovary (arrow 1) with heterogeneous signal, mostly hyper intense in T1 and T2. It fades after removal of the fat and is not diffusion-restrictive except for a small nodular area on the left anterolateral side (arrow 2) which takes on strong contrast after injection of the gadolinium chelate.

MRI was done to determine the precise characteristics of this mass. It showed a round, suprauterine mass near the left ovary, with a thin wall, containing a fatty component (T1 and T2 hyperintense, and T1 FS) with a T2 hyperintense, T1 hypointense solid component, with a high signal in diffusion-weighted imaging (DWI) and strong enhancement. MRI findings pointed to the presence of a left ovarian dermoid cyst with a potentially-malignant fleshy component classified as O-RADS MRI 5 (Fig. 2).

An exploratory laparotomy was decided. Intraoperative examination showed that the lesion was a subserosal myoma with cystic degeneration, located on the posterior wall of the uterus, and that the ovaries were normal. A myomectomy was performed.

Macroscopic examination revealed an encapsulated tumour with a smooth surface, measuring 10 × 7 × 5 cm. The cut surface was yellowish-white, with a soft consistency and without necrotic or haemorrhagic changes.

**Figure 3:**
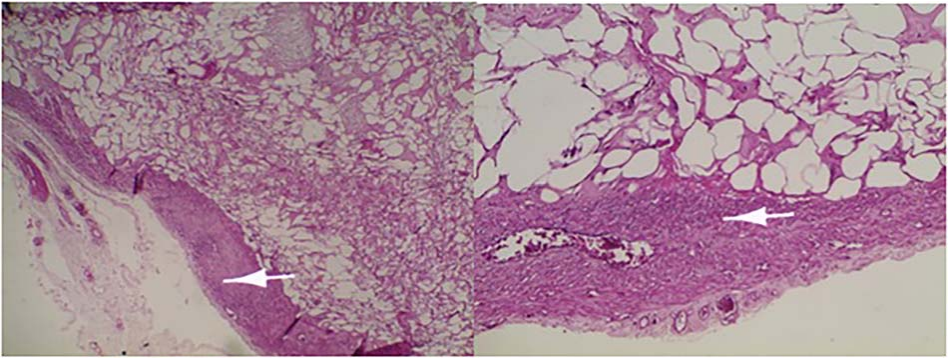
Lipoleiomyoma: proliferation consisting of a smooth muscle component visible in the periphery (arrow) and mature adipocytes separated by fibrous trabeculae. Haematoxylin and eosin; ×10.

**Figure 4:**
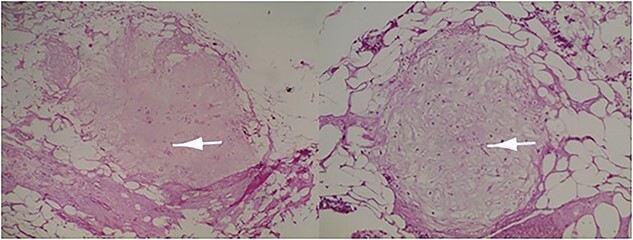
Immature or mature cartilage nodules identified in conjunction with adipocytes and smooth muscle bundles. Haematoxylin and eosin; ×20.

**Figure 5:**
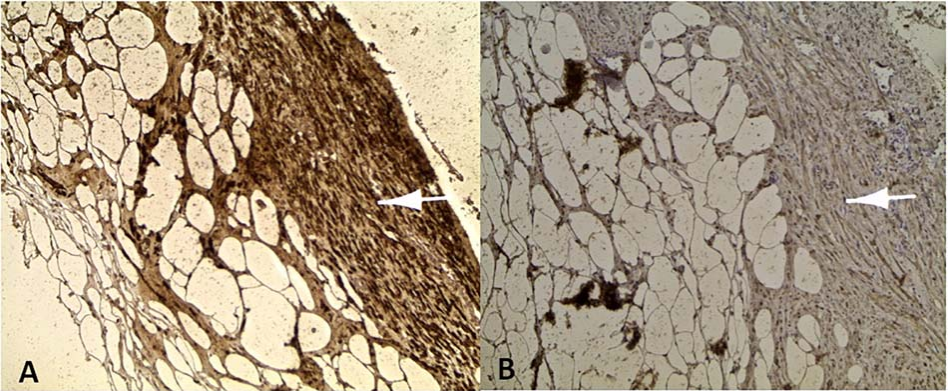
Positive labelling of smooth muscle cells with the muscle markers *Desmine* (A) and *Hcaldesmon* (B).

**Figure 6:**
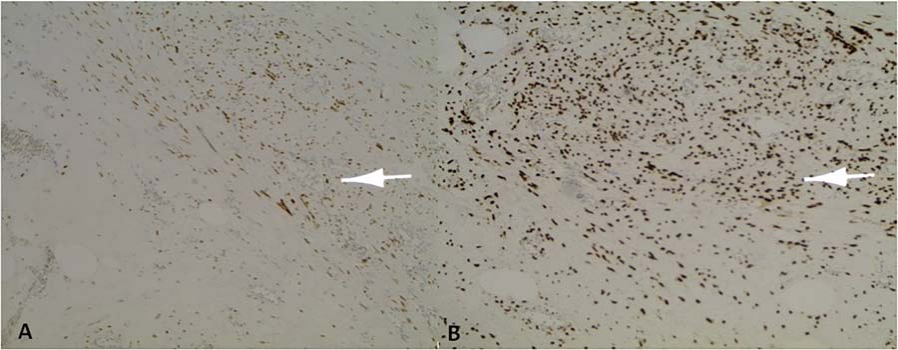
Positive nuclear labelling of smooth muscle cells with *oestrogen receptor* (ER) (A) and *progesterone receptor* (PR) (B).

Microscopic examination revealed a benign mesenchymal proliferation of fasciculated architecture within a chondromyxoid background (Fig. 3). In addition, focal areas of chondroid metaplasia were also identified (Fig. 4). On immunohistochemical analysis, the smooth muscle cells expressed desmin, h-Caldesmon, and vimentin ( Figs 5 and 6). The Ki-67 proliferation index was low.

## Discussion

Lipoleiomyomas are lipomatous variants of uterine leiomyomas that occur most commonly in postmenopausal women. Studies have shown that patients suffering from this condition often present with metabolic disorders such as diabetes, obesity, hypothyroidism, and hyperlipidaemia [4, 5]. Several theories exist. Some suggest that lipoleiomyomas may arise either from the transformation of totipotent mesenchymal cells or from the direct transformation of smooth muscle cells [4]. Oestrogen and progesterone appear to play an important role in leiomyoma cell proliferation.

Lipoleiomyomas share similar clinical features with leiomyomas, and are a variant of uterine leiomyomas [4]. Common presenting symptoms include: metrorrhagia, pain, pelvic heaviness, constipation, trouble urinating, and a palpable pelvic mass [6, 7]. In most cases, a lipoleiomyoma presents as a mass of varying size, most commonly found in the uterine corpus but can also occur in the cervix, the broad ligament, the ovary, or the retroperitoneum [4]. The differential diagnosis of a fatty pelvic mass includes a lipoleiomyoma, a benign lipoma, a liposarcoma, a mature ovarian teratoma, and benign or malignant degeneration of a leiomyoma [8].

Radiology plays a crucial role in confirming the exact location of the tumour and the presence of adipose tissue. An ultrasound scan is the first-line examination because of its non-invasiveness and availability [3]. Lipoleiomyomas usually appear as a hyperechoic, heterogeneous, avascular masses surrounded by a hypoechoic strip which represent the adjacent myometrium [4, 5]. Despite these advantages, diagnosis remains difficult because it is not always possible to determine the exact location of the lesion by sonogram alone. CT imaging allows for better detection of the fatty component of these tumours and generally shows a dense, heterogeneous, well-circumscribed mass. MRI is the imaging modality of choice for diagnostic of lipomatous tumour, as it has the ability to detect the high fat content of the tumour and accurately localize its origin. Lipoleiomyomas that are well-circumscribed on MRI have high signal intensity on T1- and T2-weighted images [9], with low signal in FAT SAT sequence [4]. Detection of fatty components of the tumour also helps to differentiate lipoleiomyomas from leiomyosarcomas [10]; however, the presence of a giant pelvic mass frequently leads physicians to suspect malignant growths of ovarian tissue or leiomyosarcoma.

Long-term follow-up of patients with uterine lipoleiomyoma has shown that these lesions are benign and that there is no recurrence or death from the disease if diagnosed as a single pelvic pathology [11] but McDonald *et al.* reported on three patients in whom liposarcomas arose from uterine lipoleiomyomas. The authors concluded that liposarcomas are likely to arise from malignant transformation of lipoleiomyomas. Nevertheless, these tumours should be added to the differential diagnosis of benign lipomatous tumours, myxoid mesenchymal tumours, and malignant mixed Mullerian tumours [12].

Despite advances, a pedunculated mass with a thin peduncle or a large mass covering the entire abdominal cavity remains difficult to diagnose using the various available radiological techniques [3], and histopathological examination remains necessary for definitive diagnosis [4]. In our case, despite the use of MRI and the fatty nature of the tumour, the pedunculated appearance that is evident intraoperatively made the lipoleiomyoma looks like a dermoid tumour even on MRI.

Histopathological analysis of the surgical specimen confirms the diagnosis by the presence of fibrotic tissue and mature adipocytes, and thus excludes other pathologies, including liposarcoma [6]. Macroscopically, these tumours are usually oval or round, sausage-shaped, solid, and multilobulated. They are moderately firm and sharply-circumscribed, with a cut surface of yellow fat that is either diffuse or localized in grey smooth muscle tissue [4]. The microscopic image is of adipocytes embedded in smooth muscle cells. The results of a study of 72 cases showed that adipose tissue and smooth muscle components express only vimentin, desmin, S100 protein, ER, PR, and Ki-67 [7].

The various heterologous elements seen in leiomyomas are fat, skeletal muscle, chondroid and osseous tissue. Metaplasia in leiomyomas is a rare phenomenon. Adipose metaplasia is the most frequently reported metaplasia. Cartilaginous metaplasia, though rare, may appear in uterine leiomyoma [2]. Our case showed multifocal areas of chondroid metaplasia.

The treatment of lipoleiomyoma depends on the age of the patient, their desire to conceive, the size and location of the tumour, the severity and type of associated symptoms. The management of small and asymptomatic lipoleiomyomas is the same as for conventional leiomyomas and is usually limited to simple expectant management [3]. Determining which option is most appropriate depends on the location and size of the tumour, the number of lesions, available surgical facilities as well as patient preference [4].

Unnecessary invasive investigations can be avoided with better understanding and improved imaging-based diagnosis of lipoleiomyomas, which can be achieved through better knowledge of their typical radiological features, particularly their fatty nature.

## Data Availability

Not applicable.
